# Effects of Dl-3-n-butylphthalide on Cerebral Ischemia Infarction in Rat Model by Mass Spectrometry Imaging

**DOI:** 10.3390/ijms18112451

**Published:** 2017-11-22

**Authors:** Run-Zhe Liu, Chao-Xin Fan, Zhi-Lin Zhang, Xin Zhao, Yi Sun, Hui-Hui Liu, Zong-Xiu Nie, Xiao-Ping Pu

**Affiliations:** 1 National Key Research Laboratory of Natural and Biomimetic Drugs, Peking University, Beijing 100191, China; 1110307320@pku.edu.cn (R.-Z.L.); fanchaoxin@bjmu.edu.cn (C.-X.F.); zhilinzhang@bjmu.edu.cn (Z.-L.Z.); zhaoxin2010@bjmu.edu.cn (X.Z.); sunyi@bjmu.edu.cn (Y.S.); 2 Department of Molecular and Cellular Pharmacology, School of Pharmaceutical Sciences, Peking University, Beijing 100191, China; 3 Key Laboratory of Analytical Chemistry for Living Biosystems, Institute of Chemistry Chinese Academy of Sciences, Beijing 100190, China; hhliu@iccas.ac.cn (H.-H.L.); znie@iccas.ac.cn (Z.-X.N.)

**Keywords:** dl-3-n-butylphthalide, permanent middle cerebral artery occlusion (pMCAO), matrix-assisted laser desorption time of flight ionization mass spectrometry imaging (MALDI–TOF–MS imaging), metabolites

## Abstract

Dl-3-n-butylphthalide (NBP) is a drug that is used in the treatment of ischaemic stroke. However, to the best of our knowledge, there are no systematic studies investigating the effects of dl-3-n-butylphtalide on the brain metabolism of small molecules. In this study, we first investigated the effects of dl-3-n-butylphthalide on the spatial distribution of small molecules in the brains of rats with permanent middle cerebral artery occlusion (pMCAO) using matrix-assisted laser desorption ionization time of flight mass spectrometry (MALDI–TOF–MS) imaging. After pMCAO modelling or a sham operation, rats were given four mg/kg of dl-3-n-butylphthalide through the caudal vein or saline once a day for nine days. The degree of neurological deficit in rats was evaluated using the modified neurological severity score (mNSS). MALDI–TOF–MS imaging was used to observe the content and distribution of small molecules related to metabolism during focal cerebral ischaemia. Multiple reaction monitoring (MRM) mode with liquid chromatography tandem mass spectrometry (LC–MS/MS) was used to verify the results obtained from MALDI–TOF–MS imaging. These small molecules were found to be involved in glucose metabolism, ATP metabolism, the glutamate–glutamine cycle, malate aspartate shuttle, oxidative stress, and inorganic ion homeostasis. Of the 13 metabolites identified by MALDI–TOF–MS imaging, seven compounds, ATP, ADP, AMP, GMP, *N*-acetylaspartic acid, ascorbic acid and glutathione, were further validated by LC–MS/MS. Taken together, these results indicate that dl-3-n-butylphthalide significantly improved ATP metabolism, level of antioxidants, and sodium-potassium ion balance in a rat model of pMCAO.

## 1. Introduction

A stroke leads to a sudden onset of cerebral circulation disorders that are primarily caused by cerebral vascular thrombosis, rupture of blood vessels, and inadequate blood or oxygen supply in brain tissue [1]. With the improvement in people’s living standards and ageing population, strokes have become a serious threat to human health, with a high incidence rate, high recurrence rate, high disability rate, and heavy economic burden. Strokes have been listed as one of the greatest challenges to human health by the World Health Organization (WHO) [2].

Based on their cause, strokes can be divided into ischaemic stroke and haemorrhagic stroke, with ischaemic strokes accounting for approximately 80–87% of all cases [3]. After an ischaemic stroke, the central part of the ischaemic brain region becomes the ischaemic core, with little blood supply, causing irreversible death of the parenchyma within a few minutes. Around the core area is the penumbra, where the nerve tissue remains at rest because of vascular bypass compensatory blood flow in this region. However, if the blood supply fails to be restored in time in the penumbra, the nerve tissue in the penumbra will eventually die and become part of the infarct [3]. The preparation of the middle cerebral artery occlusion (MCAO) by the suture method was discovered by Koizumi in 1986 [4]. After many improvements made by researchers, such as Zea-Longa and colleagues [5], it has become a common model for the study of ischaemic strokes. In this study, a modified Zea-Longa method was used to prepare the permanent middle cerebral artery occlusion (pMCAO).

**Figure 1:**
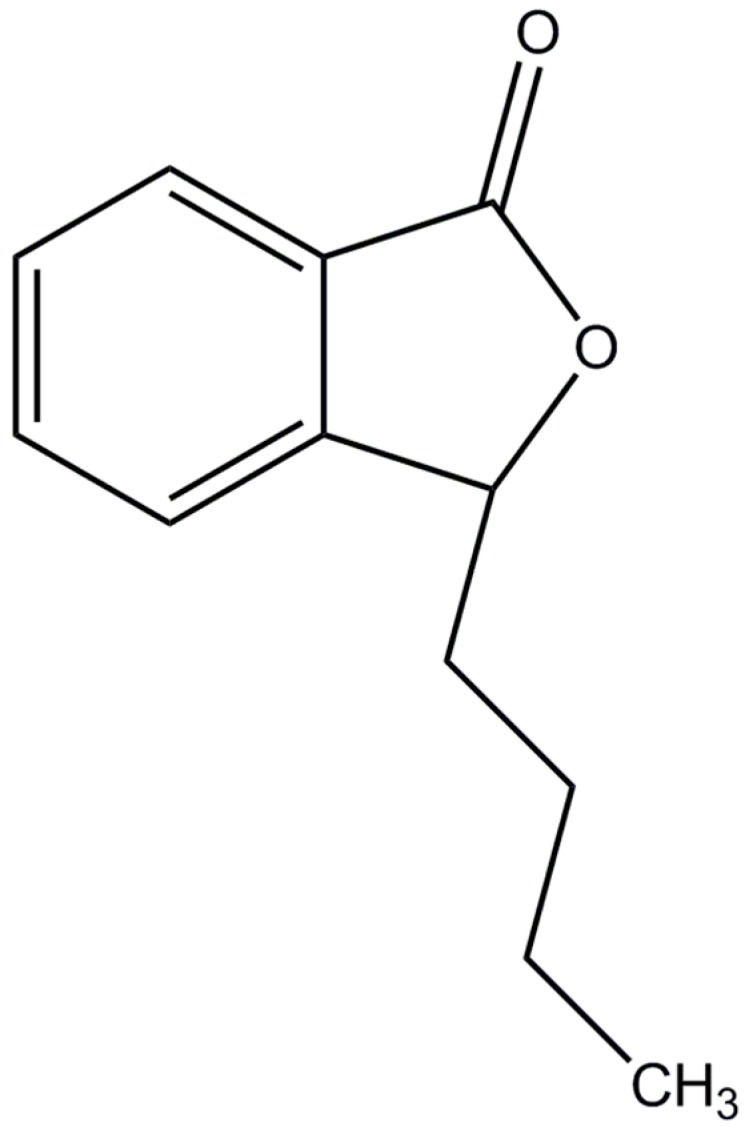
The chemical structure of 3-n-butylphthalide. Racemic 3-n-butylphthalide, or dl-3-n-butylphthalide, was used in this study.

Dl-3-n-butylphthalide (Figure 1) is a compound extracted from *Apium graveolens* Linn. Synthesized dl-3-n-butylphthalide was approved by the China Food and Drug Administration (CFDA) as a new drug for the treatment of strokes in 2002 [6], as previous studies have shown that dl-3-n-butylphthalide has beneficial anti-stroke effects, including improving cerebral blood flow [7], improving cerebral energy metabolism [8,9], reducing brain oedema [10], reducing neuronal apoptosis [11,12], decreasing focal cerebral infarction in rats [8], reducing oxidative stress [12], and inhibiting focal cerebral ischaemia inflammatory responses in rats [12]. However, there has not been a systematic study on the effects of dl-3-n-butylphtalide on brain metabolism-related small molecules, by using MALDI–TOF–MS imaging. The previous study described in reference [13,14] showed that the levels of several small molecules were altered in the brains of rats with cerebral ischaemia, as observed by matrix-assisted laser desorption ionization time of flight mass spectrometry (MALDI–TOF–MS). Therefore, our aim was to determine whether dl-3-n-butylphthalide, a common anti-stroke drug that is clinically used in China, could improve these abnormal changes.

MALDI–TOF–MS is a soft ionization mass spectrometry. The technique is simple, rapid and high-throughput with low costs and high accuracy. The principle of TOF is that ions are accelerated by the electric field to fly over the flight ducts, and the ions are detected based on the flight time of the detector. This technique is widely used for the detection of biological macromolecules, such as proteins, nucleic acids, polysaccharides and phospholipids [15,16,17,18], and based on different selections of the matrix, this method can also detect small molecules.

## 2. Results

### 2.1. Mortality

In the pMCAO group, the survival rate at the end of administration was 37.50% (6/16), which was significantly lower than that of the sham group (100%, 11/11). In the dl-3-n-butylphthalide-treated and urinary kallidinogenase-treated group, the survival rates for each group were 56.25% (9/16) and 50.00% (6/12), respectively. The Kaplan–Meier analysis indicated a difference in the survival rate between the treated groups vs. the pMCAO group, but this difference did not meet the requirement of statistical significance at *p* < 0.05 (Supplementary Figure S1 and Supplementary Table S1).

### 2.2. Modified Neurological Severity Score (mNSS)

**Figure 2:**
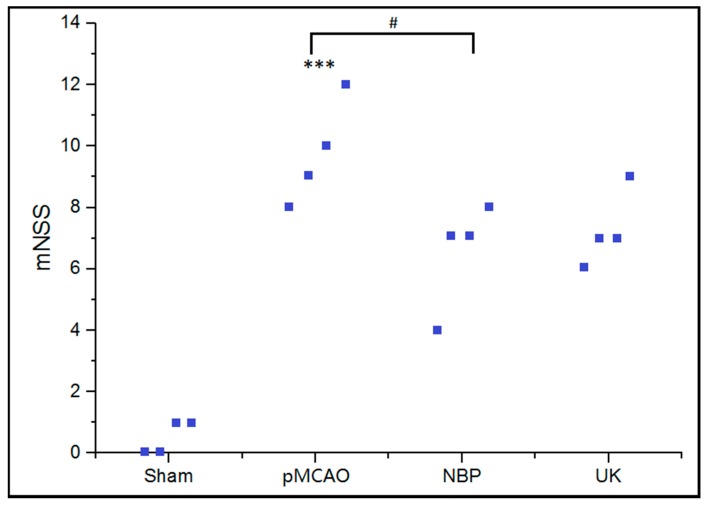
The modified neurological severity score of various groups. Sham: sham surgery group; pMCAO: permanent middle cerebral artery occlusion (pMCAO) group; NBP: dl-3-n-butylphthalide-treated group; UK: urinary kallidinogenase-treated group. *** *p* < 0.001, the pMCAO group compared with the sham group; ^#^ *p* < 0.05, the NBP group compared to the pMCAO group. *n* = 4.

As shown in Figure 2, the mNSS score was significantly higher in the pMCAO group (*p* < 0.001) compared to the sham surgery group after nine days of administration. Compared with the pMCAO group, the average score of the dl-3-n-butylphthalide treated group (4 mg/kg) obviously decreased (*p* < 0.05).

### 2.3. Dl-3-n-butylphthalide Alleviated the Abnormal Accumulation of Glucose and Citric Acid in the pMCAO Rat Model

**Figure 3:**
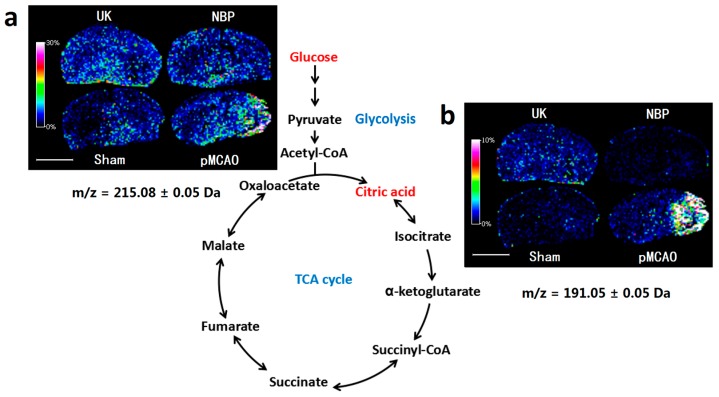
Dl-3-n-butylphthalide alleviated the abnormal accumulation of glucose and citric acid in a rat model of pMCAO. Matrix-assisted laser desorption ionization time of flight mass spectrometry (MALDI–TOF–MS) imaging of (**a**) glucose and (**b**) citric acid. The spatial resolution was set to 200 µm. *m/z*: mass-to-charge ratio. Scale bar = 5 mm. *n* = 3. Sham: sham surgery group; pMCAO: pMCAO group; NBP: dl-3-n-butylphthalide-treated group; UK: urinary kallidinogenase-treated group.

**Figure 4:**
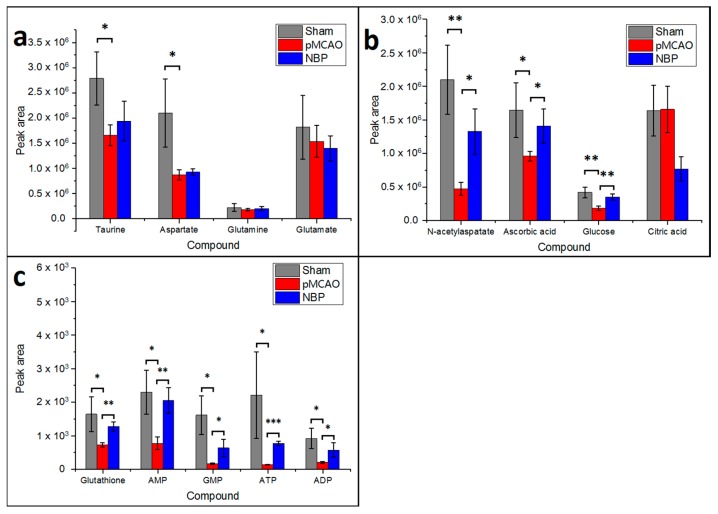
MRM MS signals of metabolites extracted from the ischaemic hemisphere of the rat brain. Data are shown as the mean ± SD (*n* = 3). The asterisks indicate significant differences (*** *p* < 0.001, ** *p* < 0.01, and * *p* < 0.05). Sham: sham surgery group; pMCAO: pMCAO group; NBP: dl-3-n-butylphthalide-treated group.

As shown in Figure 3, the glucose and citric acid contents in the right cortex and striatum of pMCAO rats increased compared with those in the sham surgery group. The glucose and citric acid contents in the right cortex and striatum decreased after nine days of treatment with dl-3-n-butylphthalide or urinary kallidinogenase. The glucose level of dl-3-n-butylphthalide was also confirmed by LC–MS/MS (Figure 4b, Supplementary Table S2).

### 2.4. Dl-3-n-butylphthalide Increased the Rate of ATP Metabolism in the Rat Model of pMCAO

**Figure 5:**
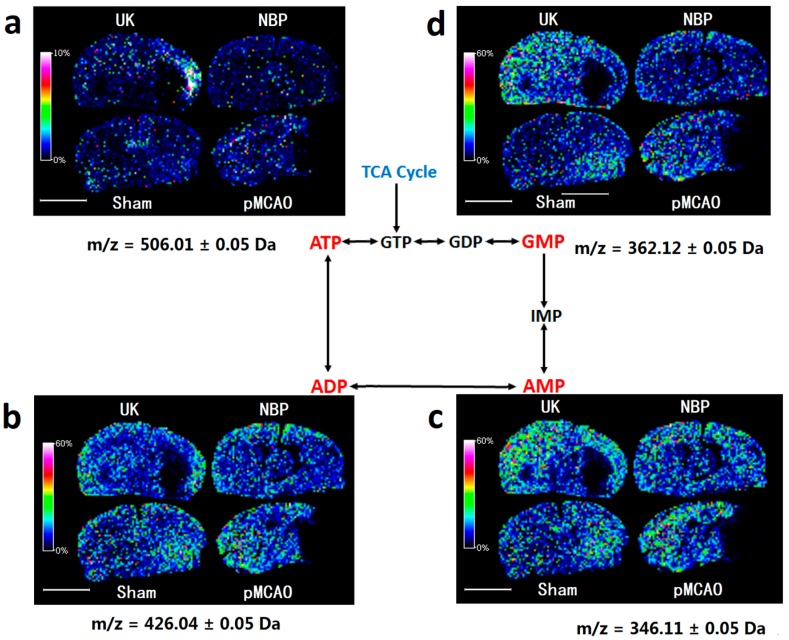
Dl-3-n-butylphthalide increased the rate of ATP metabolism in the pMCAO rat model. MALDI–TOF–MS imaging of (**a**) ATP; (**b**) ADP; (**c**) AMP; and (**d**) GMP. The spatial resolution was set to 200 µm. *m/z*: mass-to-charge ratio. Scale bar = 5 mm. *n* = 3. Sham: sham surgery group; pMCAO: pMCAO group; NBP: dl-3-n-butylphthalide-treated group; UK: urinary kallidinogenase-treated group.

As shown in Figure 5, compared with the sham surgery group, the levels of ATP, ADP, AMP, and GMP decreased in the right cortex and striatum of pMCAO rats. Dl-3-n-butylphthalide treatment increased the levels of ATP, ADP, AMP, and GMP, as revealed by MALDI-TOF-MS imaging, which was consistent with the MRM MS results from the same tissue (Figure 4c, Supplementary Table S2).

### 2.5. Dl-3-n-butylphthalide Improved the Levels of Metabolites Involved in the Glutamate-Glutamine Cycle and the Malate Aspartate Shuttle in the Rat Model of pMCAO

**Figure 6:**
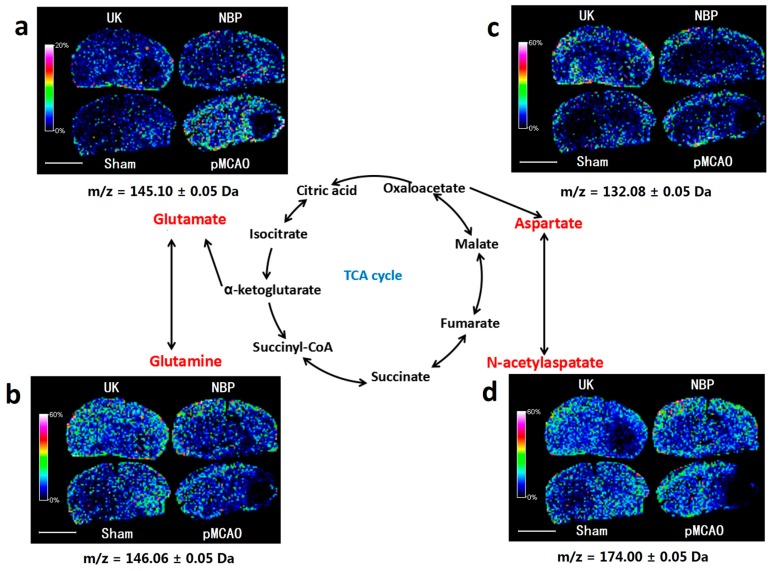
Dl-3-n-butylphthalide improved the levels of metabolites involved in the glutamate-glutamine cycle and the malate aspartate shuttle in the right cortex and striatum of pMCAO rats. MALDI–TOF–MS imaging of (**a**) glutamate; (**b**) glutamine; (**c**) aspartate and (**d**) *N*-acetylaspartate. The spatial resolution was set to 200 µm. *m/z*: mass-to-charge ratio. Scale bar = 5 mm. *n* = 3. Sham: sham surgery group; pMCAO: pMCAO group; NBP: dl-3-n-butylphthalide-treated group; UK: urinary kallidinogenase-treated group.

As shown in Figure 6, compared with the sham surgery group, the glutamic acid, glutamine, aspartate and *N*-acetylaspartate contents decreased in the right cortex and striatum of rats in the pMCAO group. Dl-3-n-butylphthalide and urinary kallidinogenase obviously increased the levels of glutamate, glutamine, aspartate, and *N*-acetylaspartate. Analysis in MRM mode of LC–MS/MS further showed relatively decreased levels of *N*-acetylaspartate at the infarct site, which dl-3-n-butylphthalide treatment further increased (Figure 4b, Supplementary Table S2).

### 2.6. Dl-3-n-butylphthalide Increased the Antioxidant Content and Improved the Balance of Metal Ions in the Rat Model of pMCAO

**Figure 7:**
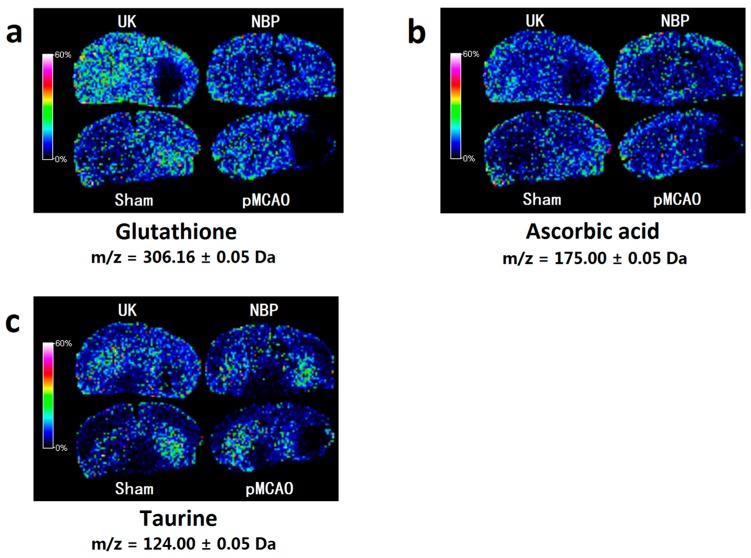
Dl-3-n-butylphthalide increased the antioxidant content in the rat model of pMCAO. MALDI–TOF–MS imaging of (**a**) glutathione; (**b**) ascorbic acid and (**c**) taurine. The spatial resolution was set to 200 µm. *m/z*: mass-to-charge ratio. Scale bar = 5 mm. *n* = 3. Sham: sham surgery group; pMCAO: pMCAO group; NBP: dl-3-n-butylphthalide-treated group; UK: urinary kallidinogenase-treated group.

As shown in Figure 7, compared with the sham surgery group, the levels of glutathione, ascorbic acid and taurine were decreased in the right cortex and striatum of rats in the pMCAO group. Dl-3-n-butylphthalide or urinary kallidinogenase increased the levels of glutathione, ascorbic acid and taurine, as indicated by MALDI–TOF–MS imaging, which was in agreement with the data obtained using the MRM mode of LC–MS/MS (Figure 4a–c, Supplementary Table S2), except that the effect of dl-3-n-butylphthalide on increasing the level of taurine was not as obvious.

**Figure 8:**
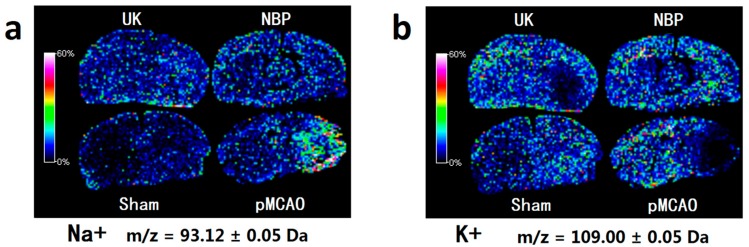
Dl-3-n-butylphthalide improved the balance of metal ions. MALDI-TOF-MS imaging of (**a**) Na^+^ and (**b**) K^+^. The spatial resolution was set to 200 µm. *m/z*: mass-to-charge ratio. Scale bar = 5 mm. *n* = 3. Sham: sham surgery group; pMCAO: pMCAO group; NBP: dl-3-n-butylphthalide-treated group; UK: urinary kallidinogenase-treated group.

As shown in Figure 8, compared with the sham surgery group, the number of sodium ions increased and number of potassium ions decreased in the pMCAO group. Dl-3-n-butylphthalide and urinary kallidinogenase reduced the number of sodium ions and increased the number of potassium ions. Dl-3-n-butylphthalide was more effective than urinary kallidinogenase in this manner.

## 3. Discussion

### 3.1. Depth of Filament Insertion

There was high mortality associated with pMCAO modelling, which increased the difficulty of the experiment. Therefore, it was important to evaluate the mortality of pMCAO by adjusting the surgical details.

During the experiment, we found that, for rats weighing 260–280 g, the mortality rate was highest within 48 h after pMCAO was established, and the mortality rate was closely related to the insertion depth of the filament into the internal carotid artery. When the filament was inserted into the internal carotid artery at a depth of 18 mm, as reported in previous studies [19], the mortality rate within 48 h of the establishment of pMCAO in rats reached 50%. Over the subsequent 10 days, rats died in succession and the mortality rate increased to approximately 70%. Even if heavier rats weighing 300–320 g were selected, the mortality rate did not decrease significantly. The mortality rate was so high that few rats survived until the end of the study. In this paper, the depth of the insertion of the thread into the internal carotid artery was set at 15–16 mm. When the insertion depth of the thread reached this level, a slight resistance was felt, at which point the insertion was stopped and the position was fixed. In this way, the mortality rate of pMCAO rats after 48 h was approximately 30%, and the total mortality was less than 50% over the following ten days. However, at the end of the pMCAO operation, some animals had no obvious symptoms, and a Longa score was used to eliminate rats with a score of less than 2. In this experiment, approximately 30% of rats were removed because of the low degree of damage caused by modelling or because they died during surgery. pMCAO rats had a high mortality rate, which was 62.5% (10/16) for the pMCAO group at 9 days after surgery. Dl-3-n-butylphthalide or urinary kallidinogenase administration reduced mortality to 43.75% (7/16) or 50% (6/12), respectively. Kaplan–Meier survival analysis (for survival functions, please refer to Supplementary Figure S1 and Table S1) showed that administration of either dl-3-n-butylphthalide or urinary kallidinogenase increased the survival rate of pMCAO rats, although the requirement of *p* < 0.05 was not met.

### 3.2. Previous Studies on Dl-3-n-butylphthalide

Extensive experimental and clinical studies have confirmed the neuroprotective effects of dl-3-n-butylphthalide, leading to the approval and marketing of dl-3-n-butylphthalide as an anti-ischaemic drug in China since 2002 [6]. Studies in animal models have suggested that butylphthalide may have neuroprotective effects [20,21,22]. Xu studied the effects of dl-, l- and d-3-n-butylphthalide on the pial arteriole diameter and blood flow velocity in focal ischaemia rats. There was a decrease in the pial arteriole diameter, and rats recovered quickly after MCAO in the dl-, l-3-n-butylphthalide-treated and nimodipine-treated groups (0.3 mg/kg), while the dysfunction of microcirculation was exacerbated by d-3-n-butylphthalide [23]. The pharmacokinetics, safety, and tolerability of l-3-n-butylphthalide tablets were established after single and multiple oral administrations in healthy Chinese volunteers. To date, dl-3-n-butylphthalide remains the only clinically approved anti-ischaemic agent in China, stressing the difficulties for a viable and effective transition from experimental to clinical practice [12]. Phase II clinical trials of dl-3-n-butylphthalide treatment in acute ischaemic stroke were approved by the FDA in 2016 [24].

### 3.3. Dl-3-n-butylphthalide Might Alleviate the Abnormal Accumulation of Glucose and Citric Acid in Rat Model of pMCAO

Glucose is the main source of energy in the brain. The TCA cycle is a key metabolic pathway that connects sugar, lipids, and protein metabolism. Citric acid is the first metabolite of the TCA cycle, which is of great significance. In this experiment, by using MALDI–TOF–MS imaging, the glucose and citric acid contents in the right cortex and striatum of pMCAO rats increased significantly compared with the sham surgery group. Dl-3-n-butylphthalide alleviated this abnormal accumulation of glucose and citric acid. There are no reports to date on the effects of dl-3-n-butylphthalide on the levels of glucose and citric acid in pMCAO rats.

The MALDI–TOF–MS and MRM MS results for some small molecules, including glucose and citric acid, were inconsistent, which may result from two reasons. First, as is seen in Figure 4 and Supplementary Table S2, most of the molecules that had insignificant results in MRM MS were molecules with lower *m*/*z* values. This may be due to endogenous brain impurities, most of which also show peaks with a low *m*/*z* value and interfere with the molecules we focused on. The existence of such impurities may affect the detection of small molecules. Another possible reason is that the MRM MS samples came from three different rats in each group, which enlarges the Standard Error of Mean (SEM). Therefore, some results, such as those for taurine, aspartate, glutamine and citric acid, which might actually be influenced by pMCAO and treatment, could not reach significance.

### 3.4. Dl-3-n-butylphthalide Increased the Rate of ATP Metabolism in the Rat Model of pMCAO

Aerobic oxidation and oxygen phosphorylation of glucose are the main forms of ATP production by organisms, and ATP can be metabolized to ADP and AMP. Energy-related metabolites are useful markers for the ongoing energy crisis during focal ischaemia. After pMCAO modelling, the decrease of cerebral blood flow reduced the oxygen supply, which blocked oxidative phosphorylation of glucose and ATP synthesis by the mitochondria. Dl-3-n-butylphthalide increased the rate ATP metabolism in the pMCAO rat model, which is consistent with the results of previous studies [9], indicating that dl-3-n-butylphthalide may have a brain protective action by increasing the rate ATP metabolism.

### 3.5. Dl-3-n-butylphthalide Might Improve the Glutamate-Glutamine Cycle in a Rat Model of pMCAO

In the TCA cycle, α-ketoacid can be converted to glutamic acid by transamination. Glutamate is an important excitatory neurotransmitter in the brain, and glutamine can also be converted to glutamate by deamination, thereby completing the glutamate-glutamine cycle. In this experiment, the MALDI–TOF–MS imaging results showed that the glutamic acid and glutamine contents decreased in the right cortex and the striatum of pMCAO rats, while dl-3-n-butylphthalide obviously increased the glutamate and glutamine levels. However, a study by Huang and colleagues [25] previously showed that dl-3-n-butylphthalide had no significant effect on the glutamate content. Similarly, the MRM MS experiment in this study showed no significant alterations of the levels of glutamate and glutamine. It is difficult to draw a strong conclusion from these disputable results, and further studies on the effects of dl-3-n-butylphthalide on glutamate are required.

### 3.6. Dl-3-n-butylphthalide Might Increase the Rate of the Malate Aspartate Shuttle in the pMCAO Rats

In addition to glutamate, another important excitatory neurotransmitter is aspartate, which has a key role in the malate-aspartate shuttle and can be synthesized from oxaloacetate and interconverted into *N*-acetylaspartate. The aspartate malate shuttle can transfer NADH from the cytoplasm to the mitochondria for oxidative phosphorylation and ATP synthesis. In this study, MALDI–TOF–MS imaging showed that the aspartate and *N*-acetylaspartate contents decreased in the cortex of pMCAO rats, while dl-3-n-butylphthalide increased the aspartate and *N*-acetylaspartate contents. The results of the MRM MS studies were consistent with those of MALDI–TOF–MS imaging for *N*-acetylaspartate, but were controversial for aspartate. No significant alterations in the levels of aspartate were present in the MRM MS results. To date, there are no reports of how dl-3-n-butylphthalide affects aspartate and *N*-acetylaspartate, so further study is needed on this topic.

### 3.7. Dl-3-n-butylphthalide Increased the Antioxidant Content

After cerebral ischaemia, the available oxygen supply is insufficient, thus reducing the synthesis of ATP, which leads to the continuous generation of reactive oxygen species, neuronal necrosis, and apoptosis. Glutathione and ascorbic acid are important antioxidants in the body. In this study, MALDI–TOF–MS imaging showed that the glutathione and ascorbic acid contents were decreased in the cortex of pMCAO rats. The glutathione and ascorbic acid contents were increased after administration of dl-3-n-butylphthalide. Previous studies have shown that dl-3-n-butylphthalide can reduce the ROS accumulation induced by hydrogen peroxide [11]. Long-term treatment with dl-3-n-butylphthalide can decrease the level of malondialdehyde in the brain tissue of rats [26]. The results indicated that dl-3-n-butylphthalide has an antioxidative effect. However, few reports have been published on the effects of dl-3-n-butylphthalide on the glutathione and ascorbic acid contents. A study by Huang and colleagues [25] reported that the taurine content in the extracellular fluid in the striatum was increased in cerebral ischaemia, while dl-3-n-butylphthalide had no significant effect on the taurine content before and after ischaemia. The MRM MS results for taurine did not agree with the MALDI–TOF–MS imaging, nor did it agree with Huang’s results. As a result, further research is needed in this area.

### 3.8. Dl-3-n-butylphthalide Maintained the Sodium and Potassium Ion Balance in the pMCAO Rat Model

Sodium and potassium ions are important for maintaining the activity of neurons. Na^+^–K^+^–ATPase is an important enzyme that is responsible for the stability of sodium and potassium ions, both inside and outside of cells. Reduced or insufficient activity of Na^+^–K^+^–ATPase is a common pathological condition during and after episodes of ischaemia. As a consequence, this condition results in the blockade of reuptake and stimulates the release of glutamate and other neurotransmitters that modulate glutamate neurotoxicity [27]. In our experiment, MALDI–TOF–MS imaging showed that dl-3-n-butylphthalide increased the concentration of potassium ions and reduced the concentration of sodium ions, which might be related to the increase in the activity of the Na^+^–K^+^–ATPase in the lumen. These results agreed well with those reported in previous studies, showing that dl-3-n-butylphthalide can improve the activity of the Na^+^–K^+^–ATPase in the mitochondria [12].

### 3.9. The Relationships among the Small Molecules

**Figure 9:**
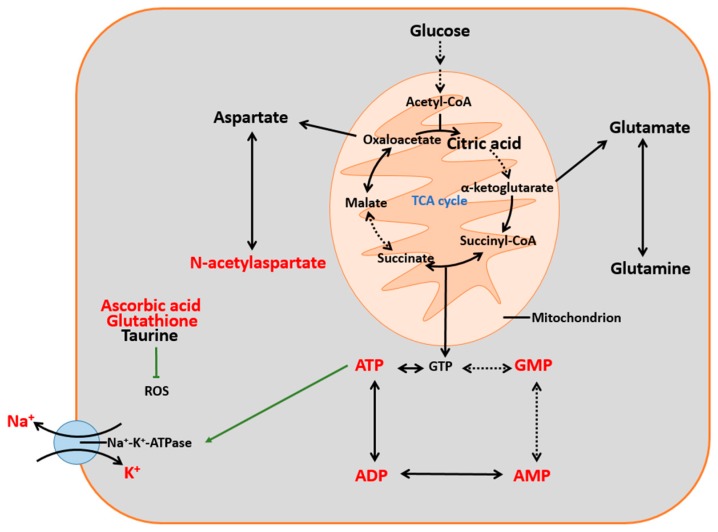
A summary of the molecules of interest in this study. Molecules in red were observed by MALDI–TOF–MS imaging and did not conflict with the results of MRM MS. Dl-3-n-butylphthalide can lower the levels of sodium ions, while increasing the levels of the other seven molecules. Black arrows indicate metabolic process. The green arrow indicates promotion, and the green T-shaped line indicates inhibition. Dashed arrows indicate omission of intermediate metabolites.

The relationships among the small molecules mentioned above are summarized in Figure 9. MALDI–TOF–MS imaging showed that dl-3-n-butylphthalide increased the levels of ATP, ADP, AMP, GMP, *N*-acetylaspartate, ascorbic acid, glutathione and potassium ions in the right cortex and striatum of pMCAO rats and decreased the sodium ion levels in the same area. These results (sodium and potassium ions excluded) were also confirmed by MRM MS, which indicated that dl-3-n-butylphthalide promoted ATP metabolism, alleviated oxidative stress and maintained the ionic balance during and after ischaemia. The other molecules of interest in this study had inconsistent results between the MALDI–TOF–MS and MRM MS tests, so further studies are necessary to draw firm conclusions concerning these molecules.

### 3.10. Dl-3-n-butylphthalide Improved the Morphology of Cerebral Coronal Sections

Cerebral coronal sections subjected to haematoxylin-eosin (HE) staining are shown in Supplementary Figure S2. Dl-3-n-butylphthalide improved the morphology of the cerebral coronal sections. In the cerebral coronal slices of pMCAO rats, some tissues were morphologically abnormal, which indicated cell death. Dl-3-n-butylphthalide treatment significantly reduced the area and severity of abnormal tissues. The histology results were consistent with the data obtained using MALDI–TOF–MS, showing that Dl-3-n-butylphthalide reduced the size of the infarct and reduced the severity of damage caused by pMCAO.

### 3.11. Semi-Quantitative Analysis of MALDI–TOF–MS

The semi-quantitative analysis of MALDI–TOF–MS is based on the same data used for MALDI–TOF–MS imaging. For MALDI–TOF–MS, the semi-quantitative results (Supplementary Figure S3A,B, Supplementary Table S3A,B) agreed with the imaging results (Figure 3, Figure 5, Figure 6, Figure 7 and Figure 8). However, the results of MALDI–TOF–MS were partially consistent with MRM MS (Figure 4) regarding the contents of ATP, ADP, AMP, GMP, *N*-acetylaspartate, ascorbic acid and glutathione. Since neither an accurate concentration nor peak area data are available for semi-quantitative analysis of MALDI–TOF–MS, peak height was selected to approximately represent the content of the molecules, and this method may not be very precise. On the other hand, after measuring the intra-day and inter-day precision of LC–MS/MS, the relative standard deviation (RSD) values should be under 15% (inter-day) and 10% (the intra-day) to meet the requirement of precision, respectively (Supplementary Table S4).

## 4. Materials and Methods

### 4.1. Animals

Adult male SD rats weighing 270–290 g and aged 8–10 weeks were purchased from Beijing Vital River Laboratory Animal Technology Co., Ltd. (Beijing, China) with the certificate number SCXK (Beijing) 2012-0001. The animals were kept in an SPF environment at the Animal Department of Peking University, sustained under standard environmental conditions (23 ± 1 °C, 45 ± 5% humidity, 12 h/12 h light/dark cycle), and fed a standard rodent diet (Keaoxieli Company, Beijing, China) with water provided ad libitum. The animals were allowed five days of acclimatization before the commencement of experimental procedures. All operations were in accordance with the requirements of the Experimentation Ethics Committee on Animal Use of the College of Medicine at Peking University in Beijing, China (approval code: LA2016159; approval date: 1 March 2016) and the United States National Academy of Sciences Guide for the Care and Use of Laboratory Animals.

### 4.2. Surgical Procedures

**Figure 10:**
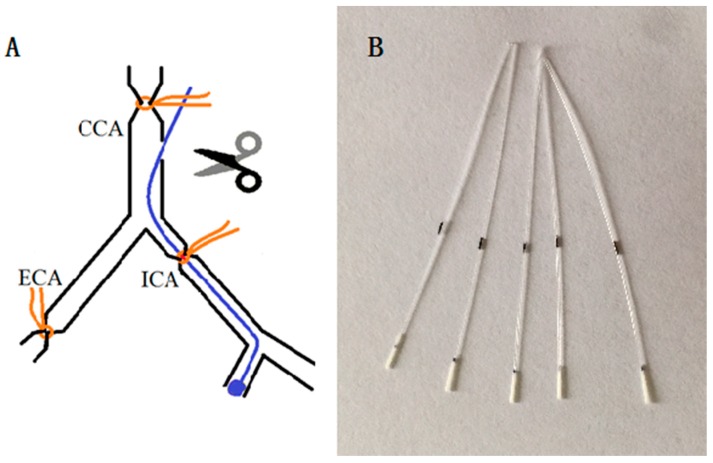
(**A**) The modelling method of pMCAO. CCA: common carotid artery; ECA: external carotid artery; ICA: internal carotid artery; Black lines represent the blood vessels, yellow lines represent the sutures, and the blue line represents the filament; (**B**) The filament used in this study. The length of the filament is 45 mm, the diameter of nylon wire is 0.28 mm, and the diameter of head end (wrapped with silastic) is 0.38 ± 0.02 mm. The mark is 20 mm to the head end.

Preparation of the pMCAO rat model was performed using the modified Longa method, as shown in Figure 10. Before the modelling operation, rats were fasted for 16 h and had free access to drinking water. Rats were first anaesthetized by an intraperitoneal injection of chloral hydrate (0.35 g/kg) at the time of operation (i.p.). Rats were fixed on a plate in the supine position, the hair of the neck was shaved, and the operation field was sterilized with 75% ethanol. An incision was made in the middle of the neck with a scalpel, through which the right carotid artery and external carotid artery were separated using ophthalmic forceps. The right external carotid artery and proximal end of the common carotid artery were ligated. A filament was inserted from a cut at the distal end of the common carotid artery. When the filament was inserted at a depth of 15–16 mm into the internal carotid artery, the resistance suddenly increased, and the insertion was stopped at that moment. The filament and distal end of the common carotid artery were fastened with sutures. The neck skin was then sutured with 75% alcohol disinfection.

Rats were woken after approximately 1 h and were then put on a spacious plane to observe their behaviour. When a rat rotated to the left, it was judged that the model was successful. Otherwise, it was considered that the model failed, and the animal was abandoned. In the sham surgery group, the vessels were separated and ligated, but vascular incision and insertion of the filament were not performed.

### 4.3. Grouping and Administration

Rats were randomly divided into four groups: the sham surgery group; pMCAO group (pMCAO); dl-3-n-butylphthalide-treated group (NBP); and urinary kallidinogenase-treated group (UK, positive drug). The total numbers of each group were 11, 16, 16, and 12, respectively, while the survival numbers before sacrifice were 11, 6, 9, and 6, for the sham, model, NBP, and UK group, respectively.

Dosage design basis: the clinical dosage of the dl-3-n-butylphthalide injection was 50 mg/70 kg/day, and the dosage of urinary kallidinogenase injection was approximately 0.15 pna/70 kg/day. According to the conversion of the body surface area between humans and animals, the rat dosage was approximately 6 times that of humans, so the rat dosage used was as follows: dl-3-n-butylphthalide at 4 mg/kg (i.v.) and urinary kallidinogenase at 0.013 pna/kg (i.v.).

Urinary kallidinogenase is a commonly used drug in the treatment of moderate strokes. Numerous studies have confirmed the efficacy and safety of human urinary kallidinogenase injection for acute ischaemic stroke [28]. Urinary kallidinogenase is a type of proteolytic enzyme extracted from human urine that can transform kininogen into kallidin and kinin, thus playing an important role in the expansion of blood vessels, promoting angiogenesis, reducing nerve cell damage, and so on [29]. Therefore, we chose urinary kallidinogenase as the positive control drug.

The 5 mg/mL injection of dl-3-n-butylphthalide was diluted to 0.5 mg/mL using saline. The urinary kallidinogenase injection was dissolved to 0.002 pna/mL using saline.

Drug administration began when rats awoke from the operation. Dl-3-n-butylphthalide and urinary kallidinogenase were delivered by a caudal vein injection. The sham surgery group and pMCAO group were given saline. Daily administration of the drugs continued for nine consecutive days. Approximately 30% of rats were eliminated because of minor injuries, and half of the other 70% of rats died during the administration period. Therefore, only 6–9 rats survived in each group that received the MCAO operation at the end of drug administration. Six rats for each group were subsequently selected for the following tests.

### 4.4. mNSS Test

The mNSS test was performed after 9 days of drug administration. The mNSS scoring system, a common standard for evaluating neurological deficits after strokes in rats, is a composite of motor, sensory, reflex, and balance tests. In this study, mNSS scoring was conducted by an investigator who was blinded to the experimental groups with Chen’s methods [30]. The score was 18 points at most, and the higher the score, the more severe the nerve injury.

### 4.5. Matrix-Assisted Laser Desorption Ionization Time of Flight Mass Spectrometry (MALDI–TOF–MS) Imaging

When the mNSS test was completed, rats were perfused with saline and their brains were snap frozen. In each group, three brain slices from different individuals were prepared for MALDI–TOF–MS imaging, and one slice was chosen to represent the results of each group.

The brain tissue was cut into one complete and smooth coronal section in the vicinity of the bregma at 0.6 mm with a 10-μm slice thickness. The slice was transferred to a special slide with an indium-tin oxide coating. The sections were dried for mass spectrometry in a vacuum pump for 30 min. Next, the tissue sections were sprayed with an ImagePrep tissue imaging matrix sprayer (Bruker Daltonics, Billerica, MA, USA). The preparation method of the matrix solution was as follows: 39.5 mg of 1,5-two amino naphthalene was added to a solution of 510 µL of 1 M hydrochloric acid and 4 mL of deionized water. The mixture was ultrasonically dissolved until the visible particles disappeared. Deionized water (2.5 mL) and high purity anhydrous ethanol (2 mL) were then added. As the matrix solutions should not be exposed to light, they were prepared immediately prior to use. The following settings were applied during the spray: spraying for 1 s, incubation for 20 s, and drying for 74 s, with 12 spraying cycles in total. The remaining parameters were set by default. After the end of a spray, the slide was sprayed a second time in the direction opposite of the previous direction so that the matrix solution was sprayed evenly.

An Ultraflextreme MALDI–TOF/TOF MS (Bruker Daltonics) equipped with a smartbeam Nd: YAG 355 nm laser was utilized for MALDI analysis. The laser was fired at a repetition rate of 2000 Hz, and the analyser was operated in negative reflector mode. The negative-ion mass spectra in the reflector mode were collected with a pulsed ion extraction time of 80 ns, accelerating voltage of 20.00 kV, extraction voltage of 17.90 kV, lens voltage of 5.85 kV, and reflector voltage of 21.15 kV. The laser spot size was set at medium focus (a laser spot diameter of approximately 50 µm), and the laser power was optimized at the start of each run and then fixed for the whole experiment. The mass spectra data were acquired over a mass range of *m*/*z* 0–1000 Da. Mass calibration was performed with external standards prior to data acquisition. For MSI analysis, the imaging spatial resolution was set to 200 µm for tissues from mice and 50 and 100 µm for brain tissues from rats. Each spectrum encompassed by 200 laser shots. The regions of interest were manually defined in the imaging software using both the optical image and MSI data image. The MALDI mass spectra were processed using total ion current (TIC) normalization, and the signal intensity of each image is represented as the normalized intensity. The results of MALDI–TOF–MS imaging were analysed according to the study by Liu and colleagues [13].

### 4.6. Multiple Reaction Monitoring (MRM) Mode of LC-MS/MS Test

Multiple reaction monitoring (MRM) was used to verify the results obtained from MALDI–TOF–MS imaging.

Sample preparation: with the exception of the urinary kallidinogenase-treated group, 1 mm-thick brain slices were cut for MRM MS, the right half of which was taken as a sample. The sample was weighed, and a 80% methanol solution was added at a proportion of 1:5 (*m*/*v*). The brain tissue was dissociated by ultrasound and centrifuged for 15 min at 10,000× *g*. The supernatant was collected. The pellet was then further extracted by a mixture of methanol and chloroform (1:1, *v*/*v*) with ultrasound. The sample was centrifuged again for 15 min at 10,000× *g*. The supernatant was combined with the previous supernatant. The sample was dried by nitrogen flow and kept at −80 °C for later use. Prior to detection, the samples were dissolved in 80% methanol and centrifuged at 18,000× *g* for 10 min. The supernatant was used for the test.

Semi-quantitative analysis of metabolites in the rat brain was performed on an AB Sciex QTrap 5500 mass spectrometer (SCIEX, Framingham, MA, USA) using multiple reaction monitoring (MRM) mode via an electrospray ionization (ESI) source in negative ion mode. A 2 µL aliquot of the sample was injected directly through a bypass into the MS without chromatography separation. The optimized instrument conditions were as follows: the electrospray voltage was maintained at minus 4500 V; turbo ion spray source temperature was set at 500 °C; nitrogen was used as the collision gas; and curtain gas (CUR), nebulizer gas (GS1), and turbo-gas (GS2) were set at 30, 50, and 50 psi, respectively. The optimized parameters, such as the quantification ion transitions and collision energy (CE) for each compound, are summarized in Supplementary Table S5. The peak area was recorded for each analyte, and data analysis was performed using the Analyst and Multiquant software programs (Applied Biosystems, Ver. 1.5.2, Waltham, MA, USA).

### 4.7. Statistical Analysis

The numerical data are presented as the mean ± SEM. The Kruskal–Wallis test was utilized in mNSS. One-way analysis of variance (ANOVA) was used to analyse the group differences according to the levels of small molecules in MRM MS. Statistical significance was set at α = 0.05 (two-sided). Survival curves were constructed using the Kaplan–Meier method. These analyses were performed using IBS SPSS Statistics (Ver. 20).

## 5. Conclusions

When observed by MALDI–TOF–MS imaging, the pMCAO operation caused significant damage to the area on the right side of the brain cortex and striatum, where the levels of a number of small molecules related to metabolism were increased or decreased, indicating severe metabolic abnormalities in the injured area. Dl-3-n-butylphthalide eased the abnormal changes in the levels of these small molecules in the ischaemic brain area, thus improving the metabolism of the ischaemic brain regions.

## Supplementary Materials

Supplementary materials can be found at www.mdpi.com/1422-0067/18/11/2451/s1.

## Abbreviations

CFDA	China Food and Drug Administration
i.p.	Intraperitoneal injection
i.v.	Intravenous injection
LC	Liquid chromatography
mNSS	Modified neurological severity scores
MALDI	Matrix-assisted laser desorption/ionization
MRM	Multiple-reaction monitoring
MS	Mass spectrometry
MSI	Mass spectrometric imaging
NBP	Dl-3-n-butylphthalide
MCAO	Middle cerebral artery occlusion
TOF	Time of flight
UK	Urinary kallidinogenase
