# Supplementary material for: Effects of Dl-3-n-butylphthalide on Cerebral Ischemia Infarction in Rat Model by Mass Spectrometry Imaging
Source: Int J Mol Sci. 2017 Nov 22;18(11):2451. doi: 10.3390/ijms18112451 (PMC5713418; doi:10.3390/ijms18112451)
Supplement: Supplementary file 1 [file ijms-18-02451-s001.pdf]

International Journal of
Molecular Sciences

Supplementary Material

Effects of Dl-3-n-butylphthalide on Cerebral Ischemia Infarction in Rat Model by Mass Spectrometry Imaging

Run-Zhe Liu[1, 2], Chao-Xin Fan[1, 2], Zhi-Lin Zhang[1, 2], Xin Zhao[1, 2], Yi Sun[1, 2], Hui-Hui Liu[4], Zong-Xiu Nie[4], Xiao-Ping Pu[1, 2, *]

1. Haematoxylin-eosin (HE) staining

Three whole brains for each group were removed and snap frozen. The section was fixed in 4% paraformaldehyde, embedded in paraffin and cut into 6 serial slices of 10 μm for HE staining. The cerebral coronal sections with HE staining were scanned under 40x magnification with an automated microscope (Motic BA600) and photographed using the Motic DSAssistant Lite software, version 1.0.

2. Preparation of the matrix

To obtain 1,5-DAN hydrochloride, 39.5 mg of 1,5-DAN was dissolved in 500 μL 1 mol/L hydrochloride and 4 mL of distilled water by sonication. Next, 4.5 mL of ethanol was added to the matrix solution.

3. Preparation of the mixture standard solution

A mixture standard solution containing taurine, L-aspartate, L-glutamate, glutamine, N-acetylaspartate, ascorbic acid, citric acid, glutathione, AMP, ADP, ATP, GMP, and glucose at 50 μM each was prepared in water.

4. Semi-quantitative analysis of MALDI–TOF–MS

For MALDI-TOF-MS, regions of interest (ROIs) (Supplementary figure 3) were defined on both the lesioned side and normal side. The average peak heights of the 15 altered metabolites in each ROI, which represented the content of the molecules, were calculated using the Bruker Daltonics flexImaging 3.0 software. The ratios of the altered metabolites on the injured side to the normal side are shown in Supplementary Table 4A, which indicates a similar conclusion to the pictured observations. The average peak heights are shown in Supplementary Table 4B.

5. Intra-day and inter-day precision of LC–MS/MS

A standard solution containing 12 components was prepared with a 20 μg/mL concentration for each component. The solution was then added to the rat brain extract that was dried under nitrogen (the rat brain extract was prepared according to the method described in section 4.6 using a normal rat brain). The mixture was centrifuged, and the supernatant was divided into 5 samples. All samples were injected once per day for three days to measure the inter-day precision. The intra-day precision was calculated based on the results of the first day. The RSD values should be under 15% to meet the requirement of precision (Supplementary Table 5).

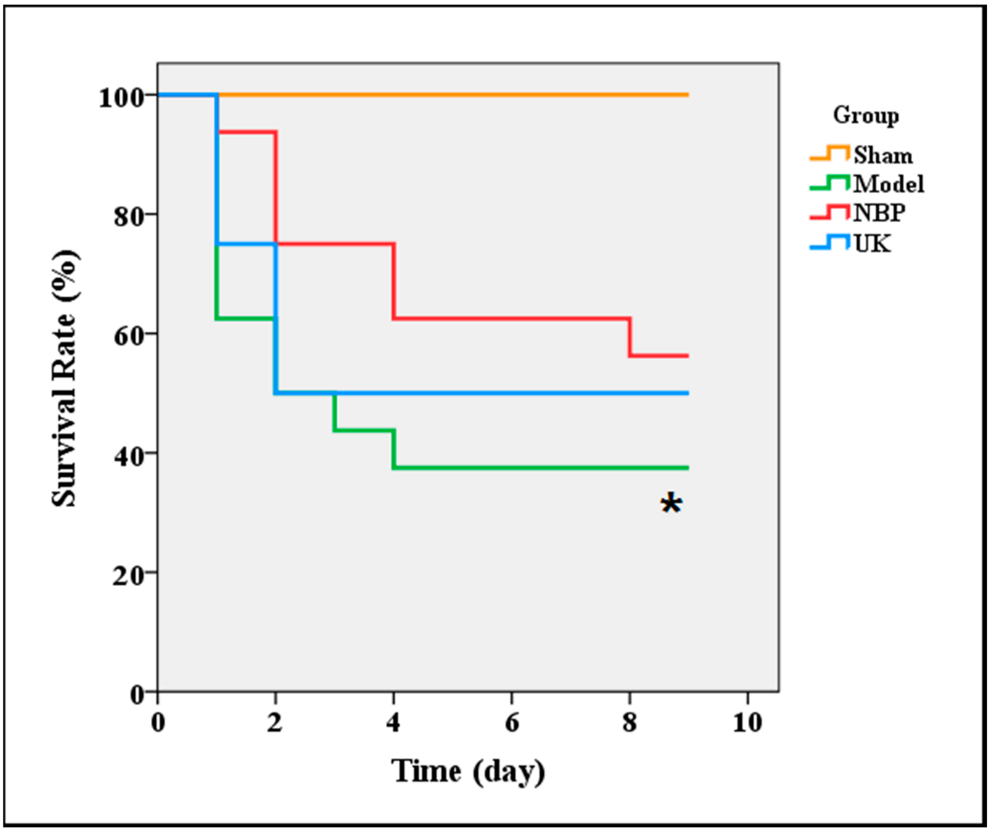

Supplementary Figure S1. Kaplan–Meier survival curves for the four groups. Sham: sham surgery group; pMCAO: pMCAO group; NBP: dl-3-n-butyphthalide treated group; UK: urinary kallidinogenase group. Data were analysed and plotted using IBM SPSS Statistics Version 20 software. * $p < 0.001$ pMCAO vs Sham.

Supplementary Table S1.

Survival rate comparison among groups.

Method	pMCAO vs Sham	NBP vs pMCAO	UK vs pMCAO
Log Rank (Mantel-Cox)	0.001	0.197	0.530
Breslow (Generalized Wilcoxon)	0.002	0.116	0.555
Tarone-Ware	0.001	0.149	0.546

Kaplan–Meier survival analysis of the four groups. Values are significance for each comparison. Log Rank, Breslow, and Tarone-Ware are 3 different analysis methods. Sham: sham surgery group; pMCAO: pMCAO group; NBP: dl-3-n-butyphthalide treated group; UK: urinary kallidinogenase group. Log Rank, Breslow, and Tarone-Ware are 3 different analysis methods.

Supplementary Table S2.

Peak areas recorded for the altered metabolites extracted from the infarct hemisphere of pMCAO rats.

	Peak area (counts)		
Analyte	Sham	pMCAO	NBP
1. Glucose (E+05)	4.23 ± 0.78	1.85 ± 0.33$^{##}$	3.53 ± 0.46*
2. Citric acid (E+06)	1.64 ± 0.38	1.66 ± 0.34	0.77 ± 0.18*
3. ATP (E+03)	2.22 ± 1.29	0.14 ± 0.01$^{##}$	0.78 ± 0.07***
4. ADP (E+02)	9.08 ± 3.09	2.08 ± 2.36$^{##}$	5.82 ± 2.12**
5. AMP (E+03)	2.30 ± 0.65	0.78 ± 0.19$^{##}$	2.06 ± 0.38**
6. GMP (E+03)	1.62 ± 0.58	0.17 ± 0.02$^{##}$	0.64 ± 0.26**
7. Glutamate (E+06)	1.82 ± 0.63	1.54 ± 0.31	1.40 ± 0.25
8. Glutamine (E+05)	2.25 ± 0.75	1.81 ± 0.23	2.05 ± 0.39
9. Aspartate (E+06)	2.10 ± 0.68	0.87 ± 0.10$^{##}$	0.92 ± 0.06
10. N-acetylaspartate (E+06)	2.10 ± 0.51	0.48 ± 0.01$^{##}$	1.33 ± 0.34*
11. Glutathione (E+03)	1.65 ± 0.05	0.73 ± 0.06$^{##}$	1.28 ± 0.14**
12. Ascorbic acid (E+06)	1.65 ± 0.41	0.96 ± 0.07$^{##}$	1.41 ± 0.25**
13. Taurine (E+06)	2.79 ± 0.53	1.66 ± 0.21$^{#}$	1.94 ± 0.40

Sham: sham surgery group; pMCAO: pMCAO group; NBP: dl-3-n-butylphthalide. Data were presented as the mean ± SD, n = 3 per group. A one-way ANOVA was used to analyse the differences between groups. $^{#}p < 0.05$, $^{##}p < 0.01$, $^{###}p < 0.001$ vs. sham group; $^{*}P < 0.05$, $^{**}p < 0.01$, $^{***}p < 0.001$ vs. pMCAO group.

Supplementary Table S3.

Semi-quantitative analysis of MALDI–TOF–MS

A. Ratios of the altered metabolites on the injured side to the normal side.

Analyte	ROI 1 / ROI 1′ (Sham)	ROI 2 / ROI 2′ (pMCAO)	ROI 3 / ROI 3′ (NBP)	ROI 4 / ROI 4′ (UK)
Na^+	140.83%	273.33%	93.82%	123.19%
K^+	156.06%	38.56%	99.07%	69.08%
Taurine	155.64%	67.90%	99.86%	91.61%
Aspartate	156.15%	53.30%	87.11%	78.47%
Glutamine	169.32%	84.75%	131.33%	118.73%
Glutamate	158.75%	55.46%	80.49%	86.34%
N-acetylaspartate	145.36%	35.36%	84.82%	59.55%
Ascorbic acid	144.68%	44.90%	83.11%	71.54%
Glucose	138.74%	1049.55%	43.32%	101.38%
Citric acid	174.75%	235.05%	89.94%	97.37%
Glutathione	155.00%	42.14%	82.48%	60.46%
GMP	135.39%	34.72%	86.74%	54.95%
AMP	166.66%	32.92%	74.39%	50.99%
ADP	129.56%	34.58%	79.89%	73.72%
ATP	83.11%	28.19%	48.56%	88.99%

Sham: sham surgery group; pMCAO: pMCAO group; NBP: dl-3-n-butyphthalide treated group; UK: urinary kallidinogenase treated group.

B. Average peak height of altered metabolites in the 8 ROIs.

Analyte	ROI 1 Sham	ROI 2 pMCAO	ROI 3 NBP	ROI 4 UK	ROI 1′ Sham	ROI 2′ pMCAO	ROI 3′ NBP	ROI 4′ UK
Na^+ (E-01)	3.83	12.71	5.83	7.02	2.72	4.65	6.21	5.70
K^+ (E-01)	5.54	2.68	5.88	5.08	3.55	6.95	5.94	7.35
Taurine (E-01)	5.01	3.35	4.37	5.65	3.22	4.93	4.38	6.16
Aspartate (E-01)	5.16	3.48	4.39	6.90	3.30	6.52	5.04	8.80
Glutamine (E-01)	1.70	3.43	2.26	2.06	1.00	4.05	1.72	1.74
Glutamate	3.11	1.39	2.29	3.27	1.96	2.51	2.85	3.79
N-acetylaspartate	15.55	5.59	14.33	9.89	10.70	15.80	16.90	16.60
Ascorbic acid (E-01)	11.16	4.32	7.95	8.30	7.71	9.62	9.57	11.60
Glucose (E-01)	1.43	23.09	0.80	3.14	1.03	2.20	1.84	3.10
Citric acid	1.58	4.55	1.76	2.29	0.90	1.94	1.95	2.35
Glutathione	2.95	1.07	1.88	2.48	1.91	2.53	2.28	4.10
GMP (E-01)	11.95	5.03	10.15	9.22	8.83	14.50	11.70	16.78
AMP (E+02)	8.66	2.82	4.23	7.19	5.20	8.57	5.69	14.10
ADP (E+01)	5.89	2.23	3.85	3.86	4.55	6.46	4.82	5.24
ATP (E+03)	3.91	2.37	4.22	8.47	4.70	8.40	8.68	9.52

Sham: sham surgery group; pMCAO: pMCAO group; NBP: dl-3-n-butyphthalide treated group; UK: urinary kallidinogenase group. AMP: adenosine monophosphate, ADP: adenosine diphosphate, ATP: adenosine triphosphate, GMP: guanosine monophosphate.

Supplementary Table S4.

Intra-day and inter-day precision of LC–MS/MS

Analyte	Intra-day (n = 5)			Inter-day (n = 3)		
	Peak area average (counts)		Precision (RSD, %) [a]	Peak area average (counts)		Precision (RSD, %) [a]
Taurine	4.45	E+07	5.32%	4.18	E+07	7.07%
Aspartate	1.83	E+07	6.99%	1.65	E+07	12.35%
Glutamine	7.40	E+06	3.98%	7.02	E+06	8.63%
Glutamate	3.09	E+07	7.67%	2.96	E+07	4.08%
N-acetylaspartate	3.33	E+07	8.02%	3.52	E+07	6.44%
Ascorbic acid	4.92	E+07	8.34%	4.85	E+07	5.31%
Glucose	5.68	E+06	9.08%	5.49	E+06	5.47%
Citric acid	1.58	E+07	8.38%	1.77	E+07	13.64%
Glutathione	3.42	E+06	8.46%	3.54	E+06	3.57%
AMP	1.01	E+04	7.95%	1.07	E+04	6.71%
GMP	1.60	E+04	6.89%	1.57	E+04	4.84%
ADP [b]	9.10	E+02	7.46%	8.70	E+02	6.69%
ATP	1.57	E+04	8.62%	1.69	E+04	5.84%

a. RSD = Standard deviation / Peak area average

b. ADP was not included in the standard solution. The ADP detected probably came mainly from rat brain extract.

Supplementary Table S5.

Multiple reaction monitoring (MRM) transitions and optimal DP and CE values for each metabolite.

Analyte	Parent ion	Product ion	DP(V)	CE (V)
Taurine	124.1	79.8	60	30
Aspartate	131.9	87.9	50	20
Glutamine	144.9	128.1	55	15
Glutamate	145.9	102.1	40	20
N-acetylaspartate	173.9	130.1	45	18
Ascorbic acid	174.9	114.9	45	18
Glucose	179.1	89.05	80	15
Citric acid	191	110.9	50	15
Glutathione	306	143.1	80	23
GMP	362.0	211.0	70	27
AMP	346.1	133.9	100	50
ADP	426.2	134.1	90	35
ATP	506.1	158.9	100	40

DP (V): declustering potential, CE (V): collision energy, AMP: adenosine monophosphate, ADP: adenosine diphosphate, ATP: adenosine triphosphate, GMP: guanosine monophosphate.

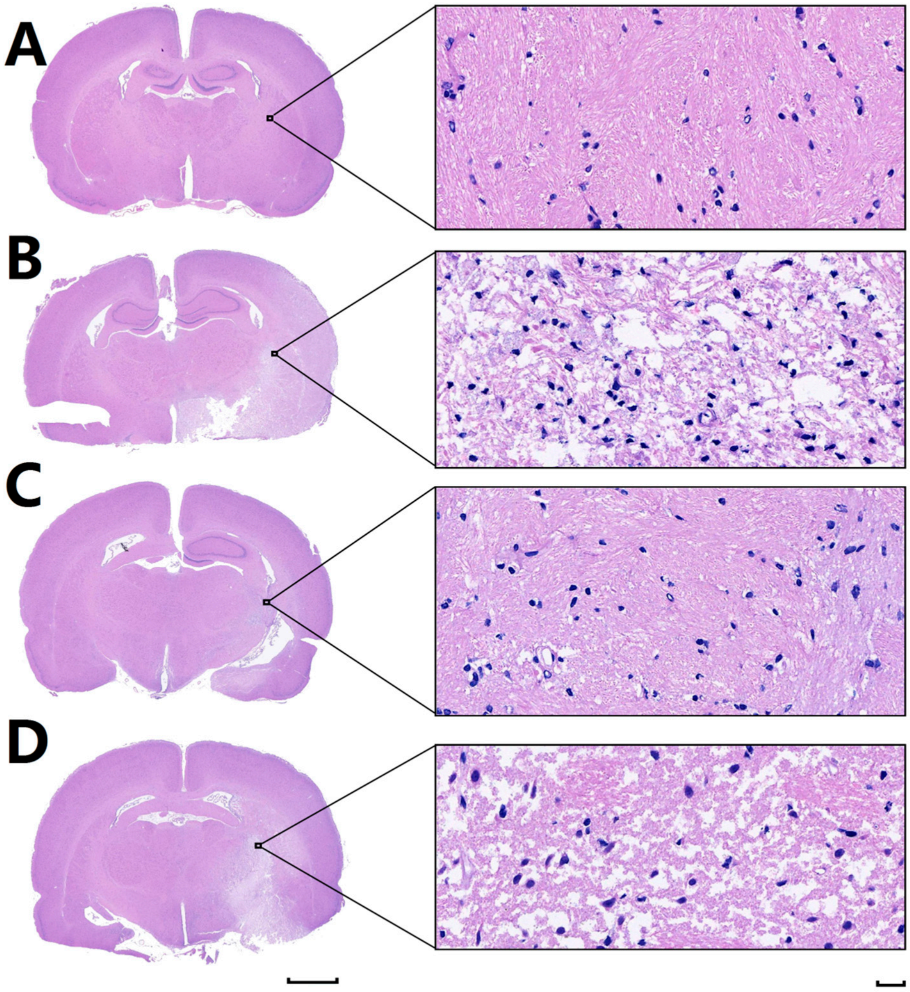

Supplementary Figure S2. Representative images of the brain morphology revealed by HE staining. (A) sham surgery, (B) pMCAO, (C) dl-3n-butylphthalide treated group and (D) urinary kallidinogenase treated group. The striatum on the lesioned side was scanned at 40x and is shown on the right. Scale bar=2 mm for the full coronal section. Scale bar = 100 μm for microscopic observation.

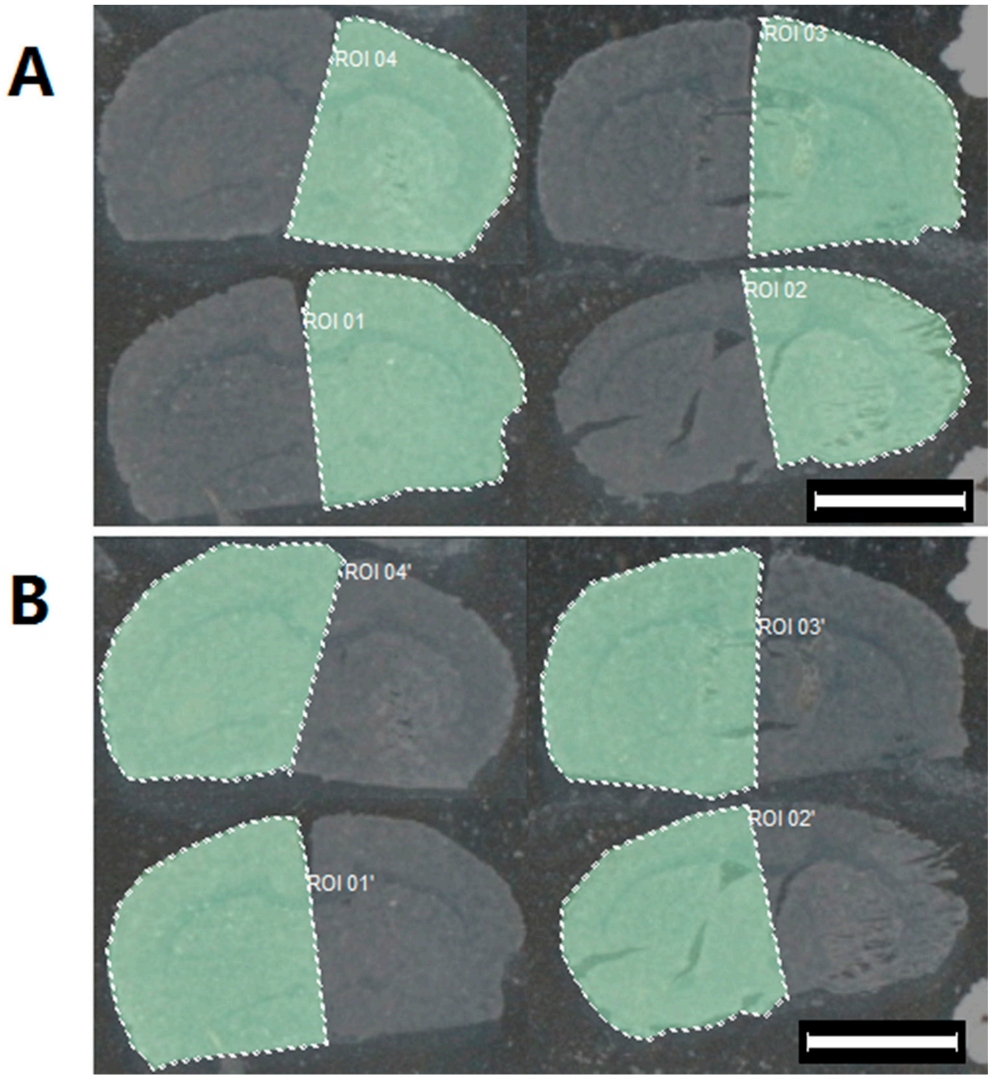

Supplementary Figure S3. Regions of interest selected for semi-quantitative analysis by MALDI–TOF–MS. (A) ROI 1 to ROI 4 are on the lesioned side of pMCAO. (B) ROI 1′ to ROI 4′ are on the normal side. The background is brain slices with the matrix sprayed. Scale bar = 5 mm.
